# Identification of potential key circular RNAs related to cognitive impairment after chronic constriction injury of the sciatic nerve

**DOI:** 10.3389/fnins.2022.925300

**Published:** 2022-08-18

**Authors:** Changliang Liu, Rui Gao, Yidan Tang, Hai Chen, Xueying Zhang, Yalan Sun, Qi Zhao, Peilin Lv, Haiyang Wang, Shixin Ye-Lehmann, Jin Liu, Chan Chen

**Affiliations:** ^1^Department of Anesthesiology, National Clinical Research Center for Geriatrics, West China Hospital, Sichuan University, Chengdu, China; ^2^Laboratory of Anesthesia and Critical Care Medicine, National-Local Joint Engineering Research Center of Translational Medicine of Anesthesiology, West China Hospital, Sichuan University, Chengdu, China; ^3^The Research Units of West China, Chinese Academy of Medical Sciences, Chengdu, China; ^4^Targeted Tracer Research and Development Laboratory, Department of Respiratory and Critical Care Medicine, West China Hospital, Sichuan University, Chengdu, China; ^5^Department of Otolaryngology, Head and Neck Surgery, West China Hospital, Sichuan University, Chengdu, China; ^6^Unité INSERM U1195, Hôpital de Bicêtre, Université Paris-Saclay, Paris, France

**Keywords:** chronic neuropathic pain, cognitive impairment, chronic constriction injury, neuroinflammation, neuronal apoptosis

## Abstract

Chronic neuropathic pain is commonly accompanied by cognitive impairment. However, the underlying mechanism in the occurrence of cognitive deficits under constant nociceptive irritation remains elusive. Herein, we established a chronic neuropathic pain model by chronic constriction injury (CCI) of the unilateral sciatic nerve in rats. Behavioral tests indicated that CCI rats with long-term nociceptive threshold decline developed significant dysfunction of working memory and recognitive memory starting at 14 days and lasting for at least 21 days. Afterward, circRNA expression profiles in the hippocampus of CCI and sham rats were analyzed *via* high-throughput sequencing to explore the potential key factors associated with cognitive impairment induced by ongoing nociception, which showed 76 differentially expressed circRNAs, 39 upregulated and 37 downregulated, in the CCI group. These differentially expressed circRNA host genes were validated to be primarily associated with inflammation and apoptotic signaling pathways according to GO/KEGG analysis and the circRNA-miRNA-mRNA network, which was also confirmed through the analysis of neuroinflammation and neuronal apoptosis. Consequently, we assumed that enhanced neuroinflammation and neuronal apoptosis might act as potential regulators of cognitive impairment induced by chronic neuropathic pain. The identification of the regulatory mechanism would provide promising clinical biomarkers or therapeutic targets in the diagnostic prediction and intervention treatment of memory deficits under neuropathic pain conditions.

## Introduction

Chronic pain, characterized by persistent pain and typical sensory, cognitive, and affective abnormalities, impacts 20∼30% of the adult population (Ta Dinh et al., 2019). Increasing clinical evidence has also demonstrated that patients with chronic pain suffer from objective adverse effects on cognition, including learning memory, decision-making, attention, processing speed, and psychomotor speed (Zhang et al., 2021). Among these, the memory processes associated with attention and working are the most likely to be affected under chronic pain conditions (Mazza et al., 2018), which may substantially influence quality of life in patients with chronic pain. Meanwhile, preclinical research has revealed that rodents with chronic neuropathic pain also develop significant damage in working memory and recognitive memory (You et al., 2018). Despite the clinical and preclinical evidence confirming that memory impairment is a frequent comorbidity in chronic pain, the underlying mechanisms remain unclear.

Alterations in mRNA expression have been validated to be correlated with a variety of diseases by regulating protein expression, including cancer, cardiovascular disease, and neurodegenerative disorders (Lee and Young, 2013). In comparison, non-coding RNAs (ncRNAs) are crucial classes of untranslated transcripts that have been proven to be a significant part of human transcriptomes and are involved in almost all physiological and pathological processes (Li et al., 2017). Circular RNAs (circRNAs), a well-established class of ncRNAs characterized by a covalently based circular structure formed by non-linear splicing, are highly expressed in mammalian brain tissues (Hanan et al., 2017). Increasing studies have revealed that circRNAs play essential roles in inhibiting the function of microRNAs, regulating the activity of RNA polymerase, and modulating the expression of corresponding genes by binding to promoters (Jens, 2014; Xiao et al., 2015). Of note, a growing number of studies have further confirmed that circRNAs are essential regulators of inflammation and apoptotic processes (Liu J. et al., 2021; Zhu et al., 2021), which are closely associated with the development of neuropsychiatric disorders. In addition, the dysregulation of circRNA expression in the dorsal root ganglion or brain could contribute to nociceptive threshold reduction and nociceptive hypersensitivity (Xin et al., 2021; Xu et al., 2021). Meanwhile, circRNAs also play substantial roles in the occurrence of cognitive impairment with different neuropathologies (Lu et al., 2019; Cao et al., 2020). However, the potential vital regulatory factors related to cognitive deficits under chronic pain conditions are extremely unclear.

Insights into the potential mechanism of long-lasting nociceptive threshold decline-induced cognitive decline promise to advance the understanding of the underlying pathophysiology and benefit from developing biomarkers and therapeutic strategies. Therefore, we fabricated a chronic neuropathic pain model by CCI on the left sciatic nerve. Behavioral tests demonstrated that the ongoing chronic pain condition of the CCI rats exhibited apparent deficits in working and recognition memory. Then, we performed transcriptome sequencing analysis to investigate the differential expression profiles of circRNAs in the hippocampus between the CCI and sham groups. The results revealed that the genes related to inflammation and apoptosis were significantly disturbed in the hippocampus of CCI rats, which indicated a possible mechanism of chronic pain-induced cognitive impairment. These results might help provide insights for clinical biomarkers and therapeutic targets for cognitive dysfunction under chronic pain conditions.

## Materials and methods

### Animals

Adult male Sprague Dawley rats (5–8 weeks, 180–220 g) were obtained from Dossy Experimental Animals Co., Ltd. (Chengdu, China) and raised under controlled conditions with a 12-h alternate circadian rhythm, a room temperature of 22–25°C, a relative humidity of 40∼70%, and food and water provided *ad libitum*. The rats were housed 2–6 per cage and allowed 1 week to acclimate to the surroundings before the beginning of experiments. All animal experiments in this study were approved by the Ethics Committee of Sichuan University and performed in strict compliance with the guidelines accepted by the International Association for the Study of Pain and the National Institute of Health Guidelines for the Care and Use of Laboratory Animals.

### Establishment of the chronic neuropathic pain model

A chronic neuropathic pain model was constructed through chronic constriction injury (CCI) of the sciatic nerve as described previously (Bao et al., 2021). Briefly, rats were anesthetized with sodium pentobarbital (50 mg/kg), and an incision was made in the left thigh to expose the sciatic nerve with almost transverse running, which was visible between the anterior and posterior groups of muscles. Four ligatures were tied loosely by chromic gut sutures 4–0 around the sciatic nerve with a 1.0∼1.5 mm interval between each ligature. The sham group was operated on by exposing only the sciatic nerve without ligation.

### Pain-related behaviors

Machinal allodynia and thermal hyperalgesia of all animals were assessed at days –1, 1, 3, 7, 14, and 21 after CCI according to previous reports (Zhang et al., 2021). Machinal allodynia was investigated by detecting the paw withdrawal thresholds (PWTs) with von Frey filaments through the up and down method. Briefly, the rats were allowed to acclimatize for 30 min in suspended cages with wire mesh floors before the test. A series of von Frey filaments weighing 0.008∼300 g was applied. The filaments were in ascending order of strength and perpendicular to the plantar surface with sufficient force to cause slight bending against the paw. Paw withdrawal, flinching, or paw licking was recorded as a positive response. Afterward, the filament of the next lower force was applied to press the plantar surface until a negative response appeared. Then, the filament of the next greater force was applied. This up-down method was repeated until five behavioral changes were determined, and the pattern of positive and negative responses was recorded. Thermal hyperalgesia was determined by detecting paw withdrawal latency (PWL) on a thermal testing apparatus (Ugo Basile plantar test, 37370, Italy). Rats were kept in transparent cages on an elevated glass plate and allowed to acclimatize for 30 min before the experiment. After that, an infrared radiant thermal stimulator was focused on the plantar surface of the hind paw through the glass plate. The time of the nociceptive endpoint was considered the PWL when the characteristic lifting or licking of the hind paw appeared. A cutoff time of 20 s was used to avoid tissue damage.

### Assessment of cognitive behavior

The cognitive function of rats after CCI was investigated through the Y maze and novel object recognition according to the literature reports (Xiong et al., 2020; Zhang et al., 2021). Prior to the cognitive behavior test, the locomotor activity of the rats was investigated through an open field test (OFT) in a large black arena of 120 × 120 × 60 cm. The total traveled distance in 5 min was recorded using a camera mounted above the arena and analyzed by the data analysis system. The OFT arena was cleaned with 75% v/v ethanol between each rat. Afterward, the Y maze spontaneous alternation test was performed to investigate exploratory behavior based on the willingness of the rats to visit a new arm rather than a familiar arm in the maze. The Y maze consists of three enclosed arms with an angle of 120 degrees, and each arm was 50 cm in length, 10 cm in width, and 25 cm in height. Each rat was gently placed in the same position of the maze and traced using a video-tracking system for an 8 min period in a caliginous and quiet room. The sequence and number of arm entries were carefully recorded, and a spontaneous alternation was defined as a rat entering all three arms on consecutive choices (ABC, ACB, BCA, BAC, CAB or CBA). The percentage of spontaneous alternation was calculated according to the formula:% spontaneous alternation = number of spontaneous alternations/(total arm entries—2) × 100%. Additionally, the novel object recognition test was performed in a box of 60 × 60 × 50 cm. The rats were placed into the box for 10 min on the first day without objects. After 2 h, two identical objects were placed into the testing box, and the rats were allowed to explore the objects for 5 min of training. After a 24 h intertrial interval in the home cage, the rats were allowed to explore the objects freely for 5 min in the same arena with one of the familiar objects and a novel object. The total time spent investigating each object within 1.5 cm using sniffing or touching the object with the nose was recorded. The longer exploration duration of a novel object was defined as evidence for intact recognition memory. The recognition index was calculated by (novel object investigation time)/(total investigation time of both objects) × 100% to compare the memory retention of each group of rats.

### Tissue collection and total RNA isolation

The rats were sacrificed at day 21 after CCI, and the hippocampus was excised quickly and stored in liquid nitrogen. Total RNA was extracted using the FastPure Cell/Tissue Total RNA Isolation Kit (Vazyme), and RNA integrity was investigated on an Agilent 2100 Bioanalyzer (Santa Clara, CA, United States). The samples with an RNA integrity number (RIN) ≥ 7 were stored at –80°C for further usage.

### RNA sequencing and analysis

Total transcriptome sequencing of six samples containing three hippocampal samples from the CCI group and three from the sham group was carried out by Shanghai OE Biotech. Co. Ltd. (China). Clean data were obtained by removing reads containing poly-N, adapters, and low-quality reads, and then the average GC content (48.51%) and Q30 base distribution (93.01∼93.4%) were analyzed. Each sample’s genome alignment was obtained with an alignment rate of 96.24∼96.63% by comparing reads to the reference genome. Afterward, the circRNA sequencing (RNA-seq) libraries were acquired, and the fold change (ratio of the average expression in each group) and *P*-value were calculated. CircRNAs with fold change ≥ 2 or ≤ 0.5 and *P*-value ≤ 0.05 were screened as the significant differentially expressed genes for further analysis. Of note, the genes undetected either in the sham groups or CCI groups were not screened in the further investigation.

### Western blot assay

When obvious cognitive impairment was exhibited at 21 days after CCI, the rats were sacrificed, and the hippocampus was excised immediately on ice. Total protein was collected from the hippocampus by homogenizing the tissues in RIPA lysis buffer supplemented with proteinase (Solarbio, Beijing, China) and phosphatase inhibitors (Solarbio). After incubation for 1 h, the protein fractions were harvested by centrifugation at 13,000 rpm for 10 min at 4°C. The protein concentrations were identified using a BCA relative protein quantification kit (Beyotime, Shanghai, China). Next, the proteins were separated in SDS-PAGE gels and transferred to poly(vinylidene difluoride) membranes (0.22 or 0.45 μm, Millipore, Bedford, MA, United States). The membranes were blocked with 5% non-fat milk (Beyotime) in Tris-buffered saline with 0.1% Tween-20 (TBST) for 1 h at room temperature. Afterward, the membranes were incubated with rabbit anti-Bax antibody (Proteintech, 50599-2), rabbit anti-Bcl2 antibody (Proteintech, 26593-1), rabbit anti-TGF-β1 antibody (Proteintech, 21898-1), rabbit anti-Cleaved Caspase3 antibody (CST, 9661), and mouse anti-α-tubulin antibody (Proteintech, 66031) overnight at 4°C. After that, the membranes were incubated with horseradish peroxidase-conjugated secondary antibodies for another 1 h at room temperature in TBST buffer containing 5% non-fat milk. Finally, the membranes were washed three times with TBST, and the blots were developed using a BeyoECL Plus Chemiluminescence Kit (Beyotime) and visualized on an Amersham Imager 600 (Cytiva, Germany). The differential expression of proteins after CCI was quantified by ImageJ software using α-tubulin as a control.

### Quantitative real-time polymerase chain reaction

Furthermore, quantification of cytokine mRNA expression and determination of the identified circRNAs in the hippocampus were investigated by quantitative real-time polymerase chain reaction (qRT-PCR). In brief, total RNA was extracted from hippocampal tissue through the TRIzol method and reverse transcribed by HiScript^○eR^ III RT SuperMix (Vazyme, Nanjing, China) according to the manufacturer’s instructions. Afterward, the templates were amplified by qRT-PCR using primers for rat genes *IL-6*, *IL-1*β, *TNF-*α, *CCL2*, *CXCR4*, *CXCL12*, *Chr6:136059672_136091144, Chr11:87902732_87908586, Chr2:104882050_104908155, Chr 17:84219927_84249246, Chr1:255706980_255774681, Chr6: 76491315_76492176, Chr8:126307382_126317853, Chr12:9 292180_9313769, Chr7:134659946_134667817, Chr10:182 18676_18238682* listed in Table 1. Each sample was run in a 20 μL reaction system of the Taq Pro Universal SYBR qPCR Master Mix kit (Vazyme). qRT-PCR was performed on a CFX96 Real-Time System (Bio-Rad, Hercules, CA, United States). Next, the relative quantification of the sample transcripts was calculated through the ^ΔΔ^Cq method with 18S as an internal reference.

**TABLE 1:** List of polymerase chain reaction primers for real-time qRT-PCR analysis.

Genes	Forward primers (5′~3′)	Reverse primers (5′~3′)
IL-6	TCTTGGAAATGAGAAAAGAGTTGTG	AGTGAGGAATGTCCACAAACTGA
IL-1β	AGCAGCTTTCGACAGTGAGG	AGGCCACAGGGATTTTGTCG
TNF-α	AGCACAGAAAGCATGATCCGA	GAAGTGGCAAATCGGCTGAC
CCL2	CTGTCTCAGCCAGATGCAGT	TGGATCTACATCTTGCATTTAAGGA
CXCR4	GCCATGGCTGACTGGTACTT	CGATGCTGATCCCCACGTAA
CXCL12	TGCCCTTCAGATTGTTGCAAGG	AGAAGCTCCAAAGCAAACCG
18S	GACACGGACAGGATTGACAG	GCTCCACCAACTAAGAACGG
Chr6:136059672_136091144	ACCCAAACATAGGTGAAGTATT	GTCTGTAGTTTCCAATATGAGG
Chr11:87902732_87908586	GCAGAATTGGCTCCATGACCT	TGTGGATGAGCTGTTGATGAAC
Chr2:104882050_104908155	AGAGGATCAGTGCTTTGACCGA	GCCTTTCATTTTCTGGGACAGT
Chr17:84219927_84249246	CCAGAAAGTGCACCGAGCT	AGCTCCATGCTTAGGTCTTCAA
Chr1:255706980_255774681	CTCGGTGCAAGCGTTGATAGAC	GCCACTGGGAGGGTAGTGTTTA
Chr6:76491315_76492176	CTCAGAACAAGATGAACCAACA	ACTCGTTAGCATAGCAGGACCA
Chr8:126307382_126317853	GTACAGGAGCCCACGGCAA	GCTGCAGCATTTTCTCCAGTTC
Chr12:9292180_9313769	ATCCAAGGCACGAAAAGCAAAG	TTTGTTTCTACCGGGCTTCCAA
Chr7:134659946_134667817	AAGAAACTGCTGACGAACTTGA	CGGCACCCTTATTTATTGACCA
Chr10:18218676_18238682	GGCAAGAGATCTTCGAGGGATT	TACCATGAGAAGGCGAGTGAGG

### Prediction of circular RNA-miRNA-mRNA association

CircRNA-miRNA interactions were predicted using circAtlas 2.0 and miRanda. The top five miRNAs were identified for each differentially expressed circRNA according to the score. Then, the network of the top five highest miRNAs connecting to one circRNA was constructed. Furthermore, we used TargetScan and miRanda databases to predict the targete genes of the miRNAs. We generally accept the overlapping genes of the two databases with the cumulative weight context++ score ≤ –0.4 and the target score ≥ 80. Then, OmicStudio tools^1^ were used to construct the circRNA-miRNA-mRNA network for the ten typical differentially expressed circRNAs that we validated as well as their predicted target genes.

### Enzyme-linked immunosorbent assay

Changes in neuroinflammation in the hippocampus after CCI were detected by Enzyme-linked immunosorbent assay (ELISA). Briefly, the hippocampi of the CCI groups and sham group at 21 days were collected on ice and homogenized in PBS. Then, the supernatant was harvested by centrifugation at 13,000 rpm for 10 min at 4°C. Thereafter, the concentrations of IL-6, IL-1β, and TNF-α in the hippocampal extract were determined with a rat IL-6 ELISA kit (ABclonal, Wuhan, China), rat IL-1β ELISA kit (ABclonal, Wuhan, China), and rat TNF-α ELISA kit (Dogesce, Beijing, China) following the manufacturer’s instructions.

### Terminal deoxynucleotidyl transferase-mediated dUTP nick-end labeling

Cell apoptosis in the central nervous system was studied through a Terminal deoxynucleotidyl transferase-mediated dUTP nick-end labeling (TUNEL) assay. Briefly, the brain tissues of the CCI and sham groups at 21 days were collected after transcardiac perfusion with 1 × PBS for 10 min and 4% paraformaldehyde for 30 min, successively. Then, the brains were excised and fixed in 4% paraformaldehyde for 48 h, followed by dehydration using sucrose containing 0.5% NaN_3_ for another 48 h. Afterward, the tissues were dissected into 10 μm thick sections and stained with a One Step TUNEL Apoptosis Assay Kit (Beyotime) following the instruction manual. Nuclei were stained with 4, 6-diamidino-2-phenylindole, dihydrochloride (DAPI) and sealed with anti-fluorescence quenching sealing solution. Apoptotic cells were observed by fluorescence microscopy (Olympus, Tokyo, Japan) at excitation wavelengths of 405 and 488 nm.

### Statistical analysis

Quantitative data are displayed as the means ± standard deviations and were analyzed using GraphPad Prism version 8.0.2 software. Student’s *t*-test was utilized as a *post hoc* test. *P* < 0.05 was identified to be statistically significant. The counts of circRNA in each sample were standardized using DESeq software, and the expression quantity was reckoned through BaseMean (Anders and Huber, 2012). Differences in circRNA expression between the sham and CCI groups were obtained by analyzing the counts of circRNAs with a negative binomial distribution test.

## Results

### Nociceptive hypersensitivity and cognitive decline after chronic constriction injury

**FIGURE 1:**
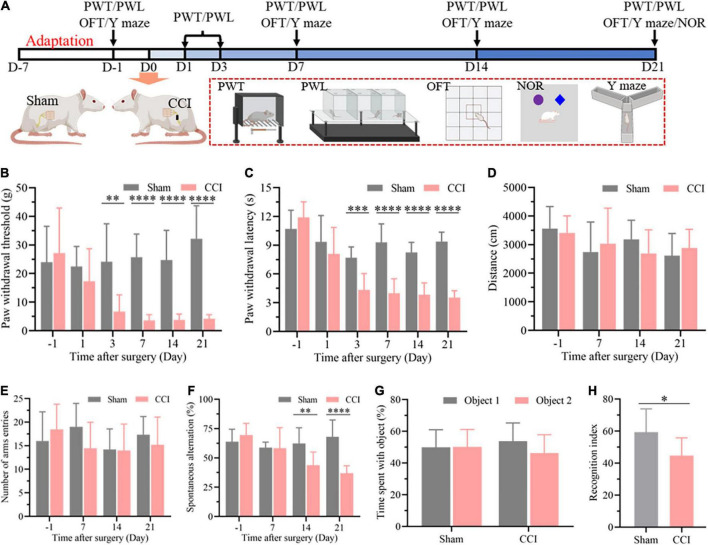
CCI contributes to nociceptive hypersensitivity and cognitive impairment. **(A)** Flowchart diagrams show the timeline of the experimental procedures. PWT represent paw withdrawal threshold; PWL represent paw withdrawal latency; OFT represent open field test; NOR represent novel object recognition. **(B)** The PWT was determined by the von Frey method. **(C)** The PWL was tested on a thermal testing apparatus within 20 s. **(D)** Locomotor activity of the rats after surgery was detected by the total travel distance in the open field. **(E)** The number of arm entries and **(F)** spontaneous alternation were detected in each group through the Y maze. Data of **(B–F)** are presented as the mean ± SD (*n* = 9), and the statistical analysis was conducted *via* unpaired Student’s *t*-test. ^**^P < 0.01, ^***^P < 0.001, ^****^P < 0.0001. **(G,H)** In the NOR test, investigation time of objects was recorded in the training and test periods, and the discrimination index was calculated in the test period. Data are presented as the mean ± SD (*n* = 12), and the statistical analysis was conducted *via* unpaired Student’s *t*-test. *P < 0.05.

As described in Figure 1A, nociceptive threshold tests were performed to evaluate allodynia in the evoked nociceptive models at 1, 3, 7, 14, and 21 days after CCI. The nociceptive thresholds were measured at 1 day before CCI as the baseline. As expected, the CCI rats exhibited a significant reduction in both PWT (Figure 1B, *P* = 0.6381 CCI vs. sham on day –1, *P* = 0.2531 CCI vs. sham on day 1, *P* = 0.0024 CCI vs. sham on day 3, *P* < 0.0001 CCI vs. sham on day 7, day 14, and day 21) and PWL (Figure 1C, *P* = 0.1755 CCI vs. sham on day –1, *P* = 0.3497 CCI vs. sham on day 1, *P* = 0.0002 CCI vs. sham on day 3, *P* < 0.0001 CCI vs. sham on day 7, day 14, and day 21) from the third day to the 21st day after surgery compared with the sham group, which demonstrated the successful establishment of a chronic neuropathic pain model in rats. Next, cognitive function was detected in the CCI and sham groups at 7, 14, and 21 days after surgery through the spontaneous alternation of rats in the Y maze (Figure 1A). Prior to the cognitive behavior test, the locomotor function of the rats after surgery was investigated through an OFT. No difference in exercise distance in this open field demonstrated the negligible influence of CCI on locomotor function (Figure 1D, *P* = 0.6520 CCI vs. sham on day –1, *P* = 0.5998 CCI vs. sham on day 7, *P* = 0.1833 CCI vs. sham on day 14, *P* = 0.4456 CCI vs. sham on day 21), which was also confirmed by the total number of arm entries without significant difference in the Y maze (Figure 1E, *P* = 0.3837 CCI vs. sham on day –1, *P* = 0.0847 CCI vs. sham on day 7, *P* = 0.9258 CCI vs. sham on day 14, *P* = 0.3787 CCI vs. sham on day 21). However, the spontaneous alternation in the Y maze was significantly decreased 14 days after CCI and until at least 21 days in comparison with the sham group at the corresponding time points (Figure 1F, *P* = 0.2590 CCI vs. sham on day –1, *P* = 0.9320 CCI vs. sham on day 7, *P* = 0.0061 CCI vs. sham on day 14, *P* < 0.0001 CCI vs. sham on day 21), indicating cognitive deficits in the CCI rats. We next performed a novel object recognition test 21 days after surgery to evaluate each group’s hippocampus-dependent memory function. In the training session, no significant difference was observed in the time spent exploring each identical object between the sham and CCI groups (Figure 1G, *P* = 0.9625 Object 2 vs. Object 1 in sham group, *P* = 0.1313 Object 2 vs. Object 1 in CCI group). In contrast, the recognition index, defined as the proportion of the amount of time spent exploring the novel object and the total time spent exploring objects, was significantly decreased in the CCI group compared with the sham group, demonstrating significant cognitive decline (Figure 1H, *P* = 0.0113 CCI vs. sham group).

### Bioinformatics analysis of differentially expressed circular RNAs in the hippocampus of chronic constriction injury rats

**FIGURE 2:**
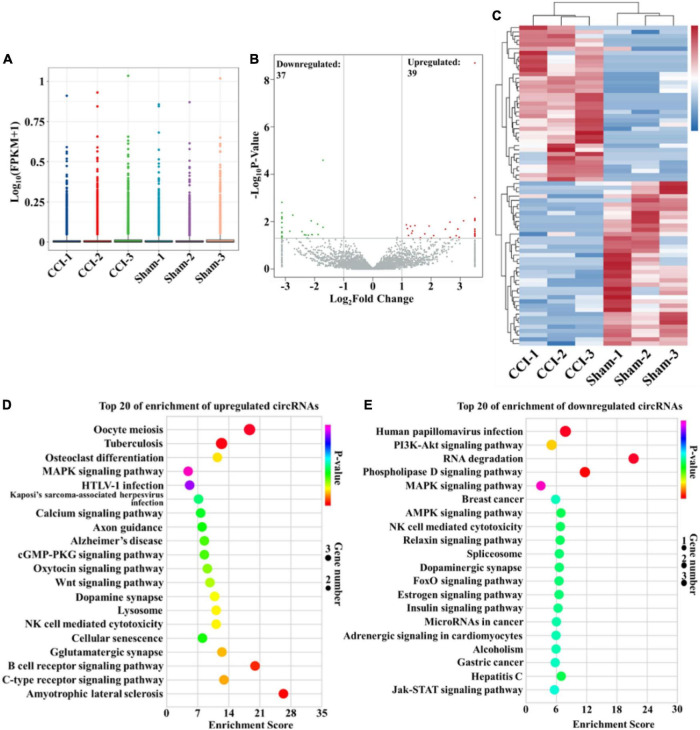
Bioinformatic analysis of differentially expressed circRNAs in the hippocampus after CCI. **(A)** Box-whisker plot showing the symmetry and distribution of data. Statistical analysis was conducted based on the minimum, first quartile (25%), median (50%), third quartile (75%), and maximum. **(B)** Volcano plots displaying the differentially expressed circRNAs with | log2(fold change)| > 1 and *P*-value < 0.05. Statistical analysis was conducted *via* a negative binomial distribution test. **(C)** Hierarchical clustering plot displaying the differentially expressed circRNA profile in the hippocampus after CCI. **(D,E)** KEGG pathway analysis showed the top 20 significantly enriched pathways and their scores for the upregulated and downregulated circRNAs. Statistical analysis was conducted *via* a hypergeometric test.

In this study, high-throughput RNA-seq was performed to profile circRNAs with differential expression in the hippocampus after CCI. The box-whisker plot was first obtained by analyzing the rat circRNA microarray, which demonstrated the good symmetry and distribution of the data (Figure 2A). Analysis of circRNA category distribution showed that the type of the identified circRNAs was mainly sense-overlapping (87.56%). In addition, antisense (1.76%), exonic (4.28%), intergenic (5.38%) and intronic (1.02%) circRNAs were also identified (Supplementary Figure 1A). Volcano plots were generated to visualize the significant differences between the sham and CCI groups with fold change ≥ 2.0 and *P*-value ≤ 0.05. According to the volcano plots, we identified 76 differentially expressed circRNAs in the two groups, including 39 upregulated and 37 downregulated circRNAs (Figure 2B and Supplementary Table 1). These differentially expressed circRNAs also primarily belonged to the sense-overlapping type with a ratio of 89.47% (Supplementary Figure 1B). Next, hierarchical cluster analysis was performed to analyze the distinguishable circRNA profiles from the hippocampal samples based on their expression levels, which indicated the different expression patterns of circRNAs in the hippocampus of CCI rats compared with those in sham rats (Figure 2C). Additionally, GO and KEGG analyses were performed using a network tool^2^ to investigate the function and signaling pathway of differentially expressed circRNA host genes. The GO analysis revealed that the significantly differentially expressed circRNAs were enriched in terms of biological process, cellular components and molecular function. These results suggested that the upregulated circRNAs had a strong relationship with the negative regulation of transcription by RNA polymerase II, cytoplasm and metal ion binding (Supplementary Figure 2A). Meanwhile, the downregulated circRNAs were associated with signal transduction, cytoplasm and ATP binding (Supplementary Figure 2B). The KEGG pathway enrichment demonstrated that the upregulated circRNAs were primarily enriched in the MAPK, cGMP-PKG and Wnt signaling pathways (Figure 2D). The downregulated circRNAs were mainly enriched in the PI3K-Akt, MAPK, AMPK, and Jak-STAT signaling pathways (Figure 2E). Of note, these processes and pathways were reported to be associated with inflammation and apoptosis in the development of cognitive deficits (Suo et al., 2018; Chen et al., 2019; Sun Q. Y. et al., 2019; Feng et al., 2020; Li et al., 2020; Liu X. Y. et al., 2021).

### Identification of differentially expressed circular RNAs

**FIGURE 3:**
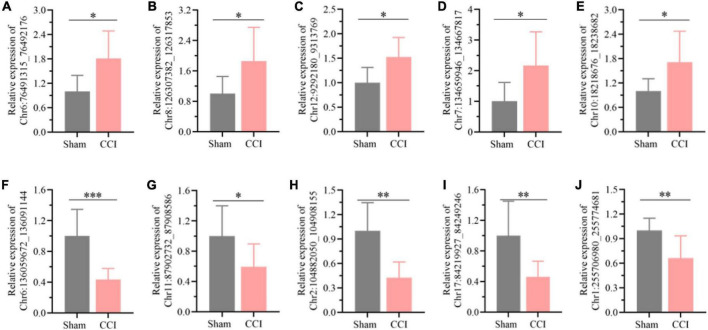
Detection of the typical differentially expressed circRNAs by qRT-PCR. **(A)** *Chr6:76491315_76492176*, **(B)** Chr8:126307382_126317853, **(C)** Chr12:9292180_9313769, **(D)** Chr7:134659946_134667817, **(E)** Chr10:18218676_18238682, **(F)** *Chr6:136059672_136091144*, **(G)** *Chr11:87902732_87908586*, **(H)** *Chr2:104882050_104908155*, **(I)** *Chr17:84219927_84249246*, **(J)** *Chr1: 255706980_255774681*. Error bars represent the mean ± SD (*n* = 8), and the statistical analysis was conducted *via* unpaired Student’s *t*-test. *P < 0.05, ^**^P < 0.01, ^***^P < 0.001.

Among all the differentially expressed circRNAs, ten typical differentially expressed circRNAs were screened and detected by qRT-PCR assay, which included *Chr6:136059672_136091144, Chr11:87902732_87908586, Chr2:104882050_104908155, Chr17:84219927_84249246, Chr1:255706980_255774681, Chr 6:76491315_76492176, Chr8:126307382_126317853, Chr12: 9292180_9313769, Chr7:134659946_134667817*, and *Chr 10:18218676_18238682.* We found that the expression of *Chr6:76491315_76492176* (Figure 3A, *P* = 0.0109), *Chr8: 126307382_126317853* (Figure 3B, *P* = 0.0284), *Chr12: 9292180_9313769* (Figure 3C, *P* = 0.0111), *Chr7: 134659946_134667817* (Figure 3D, *P* = 0.0207), and *Chr10: 18218676_18238682* (Figure 3E, *P* = 0.0268) was substantially upregulated in the hippocampus of CCI rats with cognitive dysfunction, while the expression of *Chr6:136059672_136091144* (Figure 3F, *P* = 0.0008), *Chr11:87902732_87908586* (Figure 3G, *P* = 0.0376), *Chr2:104882050_104908155* (Figure 3H, *P* = 0.0011), *Chr17:84219927_84249246* (Figure 3I, *P* = 0.0081), and *Chr1:255706980_255774681* (Figure 3J, *P* = 0.0079) was significantly decreased. The expression levels of the abovementioned circRNAs were associated with the microarray analysis results, suggesting the reliability of the microarray data.

### Circular RNA-miRNA-mRNA co-expression network for the differentially expressed circRNAs

**FIGURE 4:**
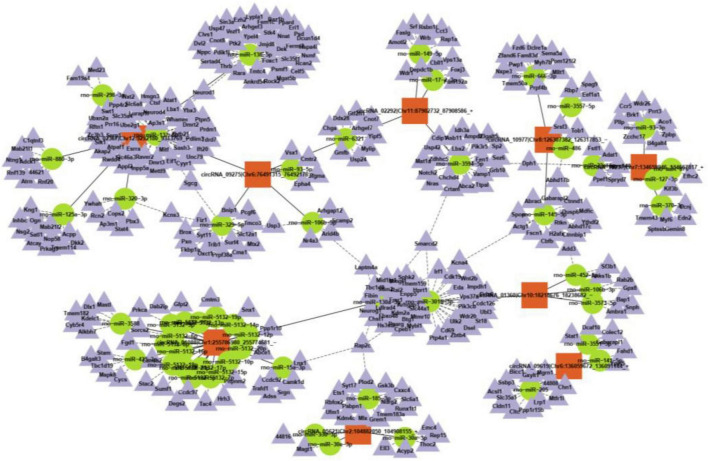
A representative circRNA-miRNA-mRNA coexpression network was obtained by OmicStudio tools.

CircRNAs can interact with miRNAs *via* miRNA response elements due to the specific binding sites of miRNAs in circRNA sequences. Five miRNAs with the highest scores among the ten typical circRNAs were predicted and are shown in Table 2. Considering that circRNAs act as miRNA sponges to regulate the circRNA-miRNA-mRNA network, we further predicted the target genes of the top five miRNAs utilizing TargetScan and miRanda. Based on the overlapping results of TargetScan and miRanda, a total of 440 target genes for the five upregulated circRNAs and 533 target genes for the five downregulated circRNAs were identified. Furthermore, the circRNA-miRNA-mRNA interaction network was constructed as shown in Figure 4.

**TABLE 2:** Predicted miRNA response elements of the ten typical differentially expressed circRNAs.

CircRNA ID	Predicted miRNA response elements (MREs)				

2-6	MRE1	MRE2	MRE3	MRE4	MRE5
Chr6:136059672_136091144	rno-miR-141-5p	rno-miR-205	rno-miR-3551-5p	–	–
Chr11:87902732_87908586	rno-miR-3594-5p	rno-miR-6321	rno-miR-149-5p	rno-let-7b-3p	rno-miR-17-2-3p
Chr2:104882050_104908155	rno-miR-330-3p	rno-miR-30e-3p	rno-miR-30a-3p	rno-miR-185-3p	–
Chr17:84219927_84249246	–	–	–	–	–
Chr1:255706980_255774681	rno-miR-130a-3p	rno-miR-5132-5p	rno-miR-3588	rno-miR-433-3p	rno-miR-15a-3p
Chr6:76491315_76492176	rno-miR-329-5p	rno-miR-106b-5p	rno-miR-137-3p	rno-miR-93-5p	rno-miR-6321
Chr8:126307382_126317853	rno-miR-145-5p	rno-miR-666-3p	rno-miR-3557-5p	rno-miR-3550	rno-miR-486
Chr12:9292180_9313769	rno-miR-880-3p	rno-miR-125a-3p	rno-miR-298-3p	rno-miR-138-5p	rno-miR-320-3p
Chr7:134659946_134667817	rno-miR-876	rno-miR-93-3p	rno-miR-127-3p	rno-miR-370-3p	rno-miR-23a-5p
Chr10:18218676_18238682	rno-miR-106b-3p	rno-miR-301b-3p	rno-miR-3573-5p	rno-miR-452-5p	–

### Evaluation of neuroinflammation in the hippocampus post chronic constriction injury

**FIGURE 5:**
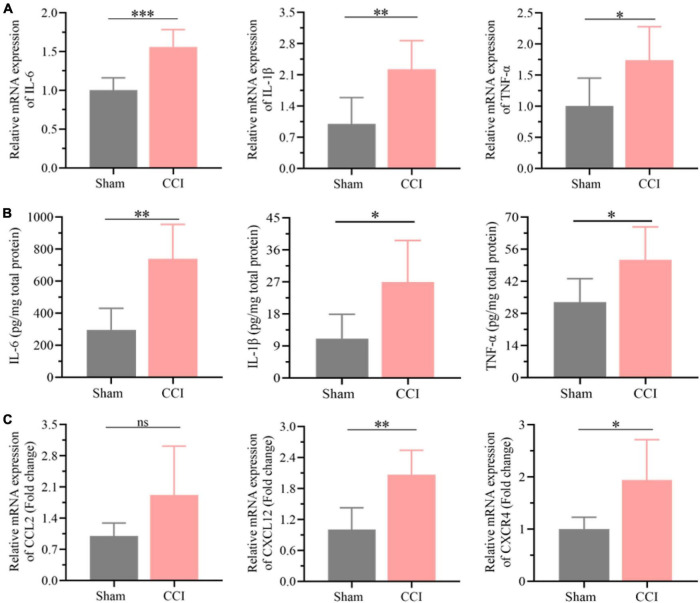
Neuroinflammation was substantially elevated in the hippocampus post CCI. **(A)** The expression levels of proinflammatory cytokines, including *IL-6*, *IL-1*β and *TNF-*α, in the hippocampus of CCI and sham rats were detected by qRT-PCR. **(B)** CCI induced upregulation of these proinflammatory cytokines in the hippocampus as detected by ELISA. **(C)** The expression levels of inflammation-related proteins, including *CCL2*, *CXCR4*, and *CXCL12*, in the hippocampus of CCI and sham rats were detected by qRT-PCR. Error bars represent the mean ± SD (*n* = 6), and the statistical analysis was conducted *via* unpaired Student’s *t*-test. *P < 0.05, ^**^P < 0.01, ^***^P < 0.001, ns represent no significance.

The results of the functional analysis revealed that neuroinflammation and neuronal apoptosis might play essential roles in the occurrence of cognitive impairment after CCI for 21 days. Thus, the key factors involved in neuroinflammation in the hippocampus of CCI and sham rats were first determined through qRT-PCR and ELISA. As expected, the expression of proinflammatory cytokines, including IL-6, IL-1β, and TNF-α, was higher in the hippocampus of CCI rats than in the sham group (Figure 5A, *P* = 0.0005 CCI vs. sham of IL-6, *P* = 0.0061 CCI vs. sham of IL-1β, *P* = 0.0275 CCI vs. sham of TNF-α), which was also validated by ELISA as shown in Figure 5B (*P* = 0.0015 CCI vs. sham of IL-6, *P* = 0.0169 CCI vs. sham of IL-1β, *P* = 0.0279 CCI vs. sham of TNF-α). In addition, in contrast with the sham group, the inflammatory chemokine C-C ligand 2 (CCL2) levels were substantially upregulated in CCI rats (Figure 5C, *P* = 0.0757 CCI vs. sham). Meanwhile, the expression of chemokine (C-X-C) receptor 4 (CXCR4) and CXCL12 was also dramatically increased with significance in the hippocampus after CCI for 21 days (Figure 5C, *P* = 0.002 CCI vs. sham of CXCL12, *P* = 0.0175 CCI vs. sham of CXCR4).

### Evaluation of neuronal apoptosis in the hippocampus post chronic constriction injury

**FIGURE 6:**
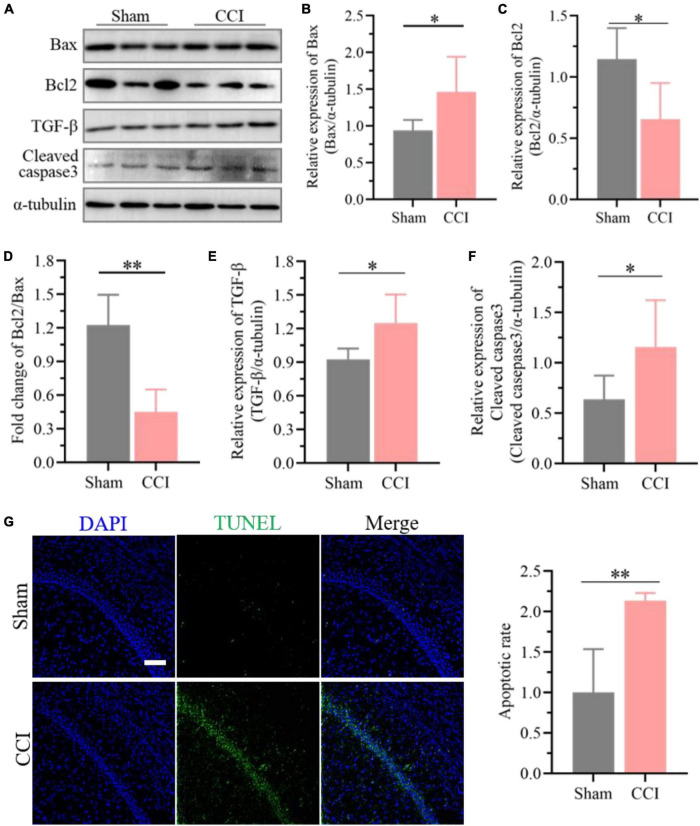
Enhanced neuronal apoptosis after CCI plays essential roles in cognitive deficits. **(A)** Representative images of western blotting showing the expression levels of Bcl-2, Bax, TGF-β and cleaved caspase-3 in the hippocampus of CCI and sham rats. **(B,C)** Quantitative analysis of **(B)** Bax and **(C)** Bcl-2 expression after CCI using ImageJ software. **(D)** The proportion of Bcl2 and Bax was calculated. **(E,F)** Quantitative analysis of **(E)** TGF-β and **(F)** cleaved caspase-3 expression after CCI using ImageJ software. Data of **(B–F)** are presented as the mean ± SD (*n* = 6), and the statistical analysis was conducted *via* unpaired Student’s *t*-test. *P < 0.05, ^**^P < 0.01. **(G)** Representative images for TUNEL detection in the hippocampal CA1 region after CCI, and the percentage of apoptotic ratio was quantified. Error bars represent the mean ± SD (*n* = 5), and the statistical analysis was conducted *via* unpaired Student’s *t-*test. ^**^P < 0.01. Scale bar: 100 μm.

Furthermore, the expression of Bcl-2 was significantly decreased in the hippocampus of CCI rats at 21 days (Figures 6A,C, *P* = 0.0116 CCI vs. sham), while Bax in the hippocampus was upregulated substantially (Figures 6A,B, *P* = 0.0275 CCI vs. sham), and the proportion of Bcl-2 and Bax further declined significantly (Figure 6D, *P* = 0.0002 CCI vs. sham). Meanwhile, western blotting results demonstrated that ongoing chronic neuropathic pain stimuli resulted in the upregulation of TGF-β (Figures 6A,E, *P* = 0.0160 CCI vs. sham) and cleavage of caspase-3 (Figures 6A,F, *P* = 0.0339 CCI vs. sham). Additionally, the ratio of apoptotic neurons in the hippocampal CA1 region of CCI rats was markedly improved compared to that of sham rats according to the TUNEL staining assay (Figure 6G, *P* = 0.0016 CCI vs. sham).

## Discussion

Cognitive impairment is one of the most common complications associated with chronic pain, which was also validated in preclinical observations of chronic neuropathic pain in rodents (Mazza et al., 2018; Zhang et al., 2021). However, the underlying mechanisms of chronic pain-induced memory dysfunction are still indistinct. In the present work, we established chronic neuropathic pain models by CCI of the sciatic nerve. Behavioral test results indicated that CCI rats with long-term nociceptive threshold reduction developed typical cognitive deficits in working memory and recognition memory. There was no substantial difference in the total traveled distance in the OFTs after surgery, which demonstrated that recognitive impairment was unlikely to be connected with deficits in locomotor function and exploratory activities during recognitive test periods. Previous reports have validated that nociceptive hypersensitivity induced by CCI always occurs 2 days after surgery, reaches a plateau at 6∼7 days and is constantly sustained for at least 30 days (Bridges et al., 2001), which is consistent with our results. Additionally, our results also suggested that the cognitive function of CCI rats decreased significantly 14 days after surgery and lasted until 21 days. Thus, we collected hippocampal tissues on day 21 for further transcriptome sequencing and identified the potential key factors associated with cognitive impairment.

CircRNAs are endogenous non-coding RNAs with covalently closed loops that endow them with higher stability than linear RNAs and sequence conservatism among species (Zhang et al., 2019). Most circRNAs have been determined to be more abundant in the mammalian brain than in other tissues, demonstrating that circRNAs may play a vital role in the pathological process of some neurological disorders (Hanan et al., 2017). For instance, circRNA has been confirmed to mediate synaptic and amyloid precursor protein processing deficits through the circHDAC9/mi-138/sirtuin-1 pathway in Alzheimer’s disease (Lu et al., 2019). Aerobic exercise could improve vascular cognitive impairment through circRIMS2/miR-186/BDNF-mediated neuronal apoptosis (Niu et al., 2021). Moreover, increasing evidence has suggested that neuropsychiatric disorder-associated circRNAs in the blood and CSF are promising non-invasive biomarkers for the diagnosis of complex neuropsychiatric disorders (Zhuo et al., 2020). However, the clinical value and roles of circRNAs in neuropathic pain-induced cognitive impairment are still unclear. Therefore, we performed microarray analysis for circRNAs in the hippocampus of CCI and sham rats to explore the potential key circRNAs with a significant connection to cognitive impairment. A total of 76 differentially expressed circRNAs were identified in the hippocampus between CCI and sham rats, including 39 upregulated and 37 downregulated circRNAs. Afterward, we verified ten circRNAs during the validation process, which are all consistent with the microarray results. Notably, the pathogenesis of cognitive impairment is complicated, and one signaling pathway might be regulated by a series of upstream molecules, such as RNAs. Thus, the function of the differentially expressed circRNAs was further investigated in the present work.

Generally, circRNAs serve functions through competing endogenous RNAs or miRNA sponges, interacting with RNA binding proteins, modulating the stability of mRNAs, regulating gene transcription and translating proteins (Meng et al., 2017). Therefore, the functions and associated signaling pathways were further analyzed through GO and KEGG enrichment. The GO analysis showed that the differentially expressed circRNAs were involved in dendritic spines in terms of cellular comments. Dendritic spines are post-synaptic structures at a majority of excitatory synapses in the mammalian brain, whose number and size are closely related to cognitive function in different neurological diseases (Herms and Dorostkar, 2016; Zhang et al., 2020). Moreover, transcription and ATP-associated functions in terms of biological processes and molecular functions were also enriched in GO analysis and are also involved in cognitive impairment by regulating inflammatory and apoptotic reactions (Sun X. Y. et al., 2019; Velasquez et al., 2019). Meanwhile, the KEGG enrichment analysis indicated that the upregulated circRNAs were mainly enriched in the MAPK, cGMP-PKG and Wnt signaling pathways, and the downregulated circRNAs were primarily enriched in the PI3K-Akt, MAPK and AMPK signaling pathways. These signaling pathways have been validated to be closely related to inflammation and apoptosis (Suo et al., 2018; Chen et al., 2019; Gabbouj et al., 2019; Sun Q. Y. et al., 2019; Li et al., 2020; Yue and Lopez, 2020; Liu X. Y. et al., 2021), further indicating that neuroinflammation and apoptosis might play vital roles in the development of cognitive deficits after CCI. Additionally, the miRNAs and targeted genes associated with the typical upregulated and downregulated circRNAs were further identified. Further analysis of the circRNA-miRNA-mRNA network also demonstrated that several miRNAs and target genes were closely related to inflammation and apoptosis in the development of neurodegenerative disease.

As expected, the expression of proinflammatory cytokines such as IL-6, IL-1β, and TNF-α and the inflammatory chemokine CCL2 in the hippocampus increased dramatically after CCI, suggesting elevated neuroinflammation in the hippocampus. A previous study reported that overexpressed CXCL12 could lead to memory dysfunction in neuropathic pain by recruiting inflammatory monocytes into the perivascular space (Mai et al., 2021). Interestingly, the expression of CXCR4 and CXCL12 was also higher in the hippocampus of CCI rats than in sham rats, which further demonstrated improved inflammation in the hippocampus. Bcl-2 and Bax have been proven to play essential roles in the process of apoptosis (Opferman and Kothari, 2018) and are extremely disturbed in the hippocampus after CCI. Meanwhile, the expression of activated caspase-3 and TGF-β was also increased substantially, further indicating that neuronal apoptosis occurring in the hippocampus was associated with the development of cognitive dysfunction after CCI. Consequently, improved neuronal apoptosis might be a key potential factor in neuropathic pain-induced cognitive dysfunction, which was also confirmed by TUNEL staining. Therefore, it could be hypothesized that the dysregulation of circRNA expression in the hippocampus after CCI-induced long-lasting nociceptive threshold reduction may contribute to cognitive dysfunction by improving neuroinflammation and neuronal apoptosis.

Our study also has several potential limitations. In the present work, we provide an expanded perspective on possible circRNAs and a potential mechanism promoting cognitive deficits induced by chronic neuropathic pain. However, the expression of the differentially expressed circRNAs was only detected in the hippocampus at one time point after CCI when learning and memory dysfunction was observed. Further verification of these screened circRNAs at several time points in CCI and other chronic neuropathic pain models need to be performed to provide more clinical significance for diagnosis or prediction. Additionally, the possible roles of circRNA in CCI-induced cognitive impairment were based on bioinformatics prediction. Therefore, further *in vivo* investigation should be performed to identify how these differentially expressed circRNAs initiate cognitive deficits in CCI. Meanwhile, more experiments should be carried out to determine the changes in the potential key circRNAs in different structures, including the dorsal root ganglion and dorsal root of the spinal cord, and to further explore the association of the identified circRNAs between cognitive impairment and chronic neuropathic pain. Besides, the association between the differentially expressed circRNAs and cognitive impairment induced by chronic pain might be a concomitant phenomenon. These differentially expressed circRNAs might be involved in the regulation of some other pathophysiological processes. In addition to cognitive dysfunction, affective disorders such as anxiety and depression are serious adverse consequences of chronic pain (Haanpaa et al., 2011; Radat et al., 2013). Previous evidence has demonstrated that more than 60% of chronic pain patients suffer from anxiety or depression (Kremer et al., 2021), which has also been observed in different rodent models of chronic pain (Mogil, 2009). Moreover, increasing evidence has also confirmed that inflammation and apoptosis in the hippocampus play vital roles in the development of mood disorders (Zhou et al., 2018). Thus, further studies are needed to identify the potential regulatory effects of the differentially expressed circRNAs in the hippocampus in the occurrence of mood disorders.

## Conclusion

In conclusion, our study confirmed the significant differentially expressed circRNAs and predicted miRNAs and genes involved in the development of cognitive impairment induced by chronic neuropathic pain. This study provides a preliminary perspective for circRNAs with different expression levels and predicts their potential participation mechanisms in cognitive deficits. Indeed, further studies are still needed to investigate how these circRNAs function on genes regulating cognitive dysfunction and become novel potential peripheral clinical biomarkers and therapeutic targets for constant nociceptive irritation-triggered cognitive impairment.

## Data availability statement

The datasets presented in this study can be found in online repositories. The names of the repository/repositories and accession number(s) can be found below: NCBI BioProject, and the NCBI accession number is PRJNA796160.

## Ethics statement

The animal study was reviewed and approved by the Ethics Committee of Sichuan University.

## Author contributions

CL proposed the project, designed and performed the major experiments, and wrote the manuscript. JL and CC supervised the project and provided funding. RG, YT, HC, XZ, SY-L, QZ, PL, HW, and YS participated in some experiments and data analysis. All authors listed have made a substantial, direct, and intellectual contribution to the work, and approved it for publication.

## Footnotes

1
https://www.omicstudio.cn/tool/56

2
https://cloud.oebiotech.cn/task/
