# Supplementary figures and images for: Identification of potential key circular RNAs related to cognitive impairment after chronic constriction injury of the sciatic nerve
Source: Front Neurosci. 2022 Aug 18;16:925300. doi: 10.3389/fnins.2022.925300 (PMC9433970; doi:10.3389/fnins.2022.925300)

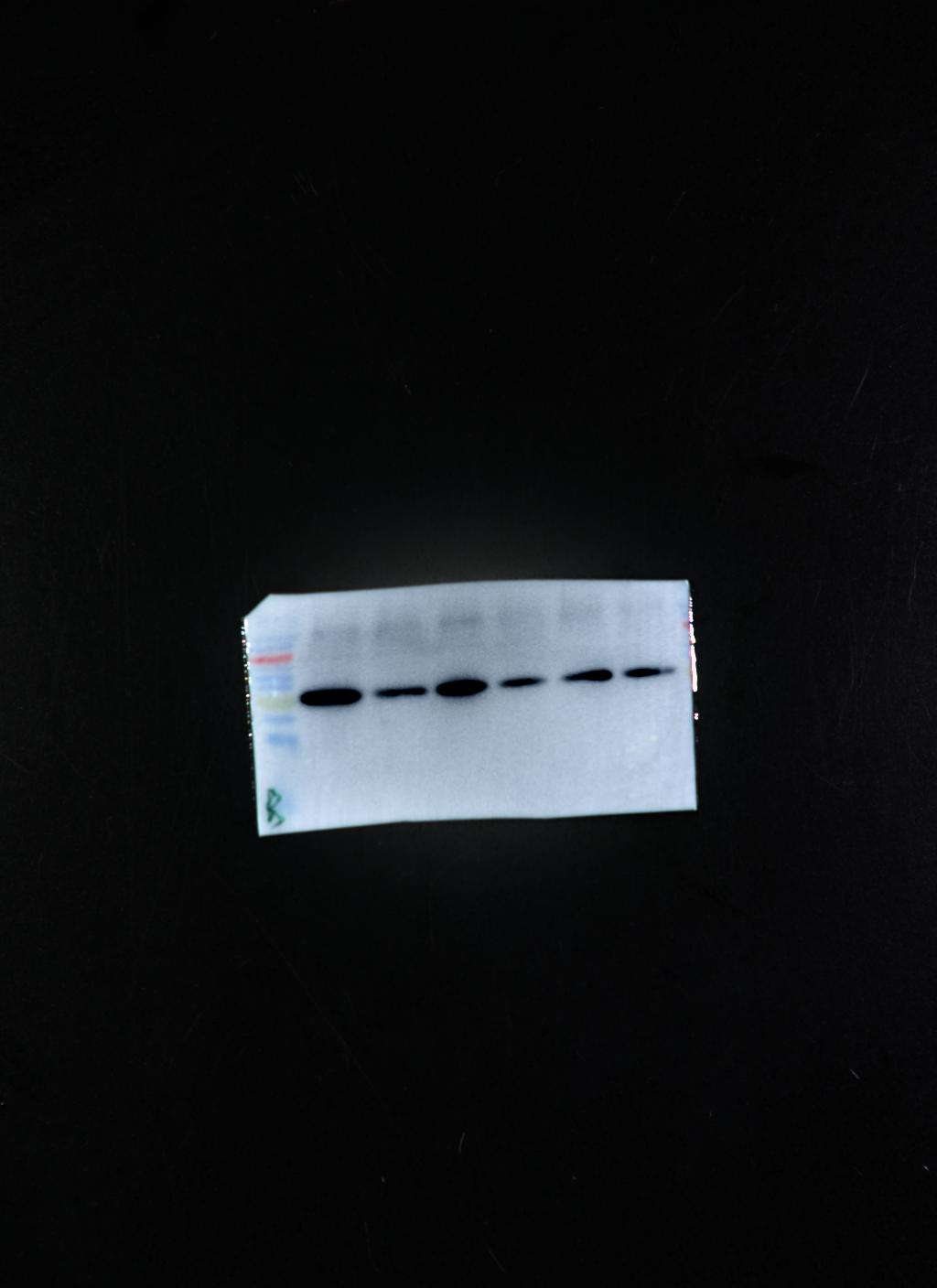

Supplement: Supplementary file 1 [file Data_Sheet_1.ZIP › Raw data/Figure 6/A. BCL2 Sham-CCI.jpg]

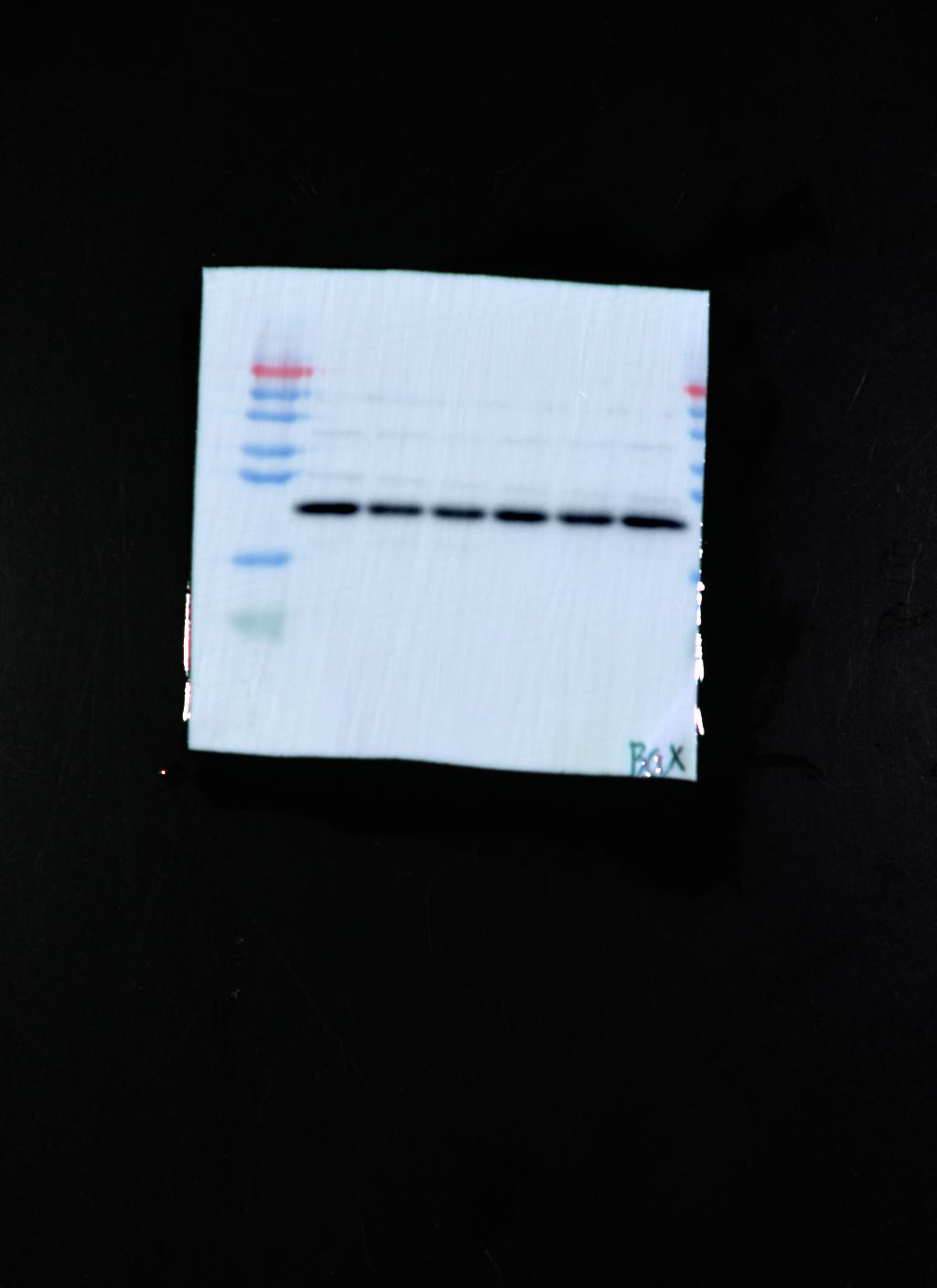

Supplement: Supplementary file 1 [file Data_Sheet_1.ZIP › Raw data/Figure 6/A. Bax Sham-CCI.jpg]

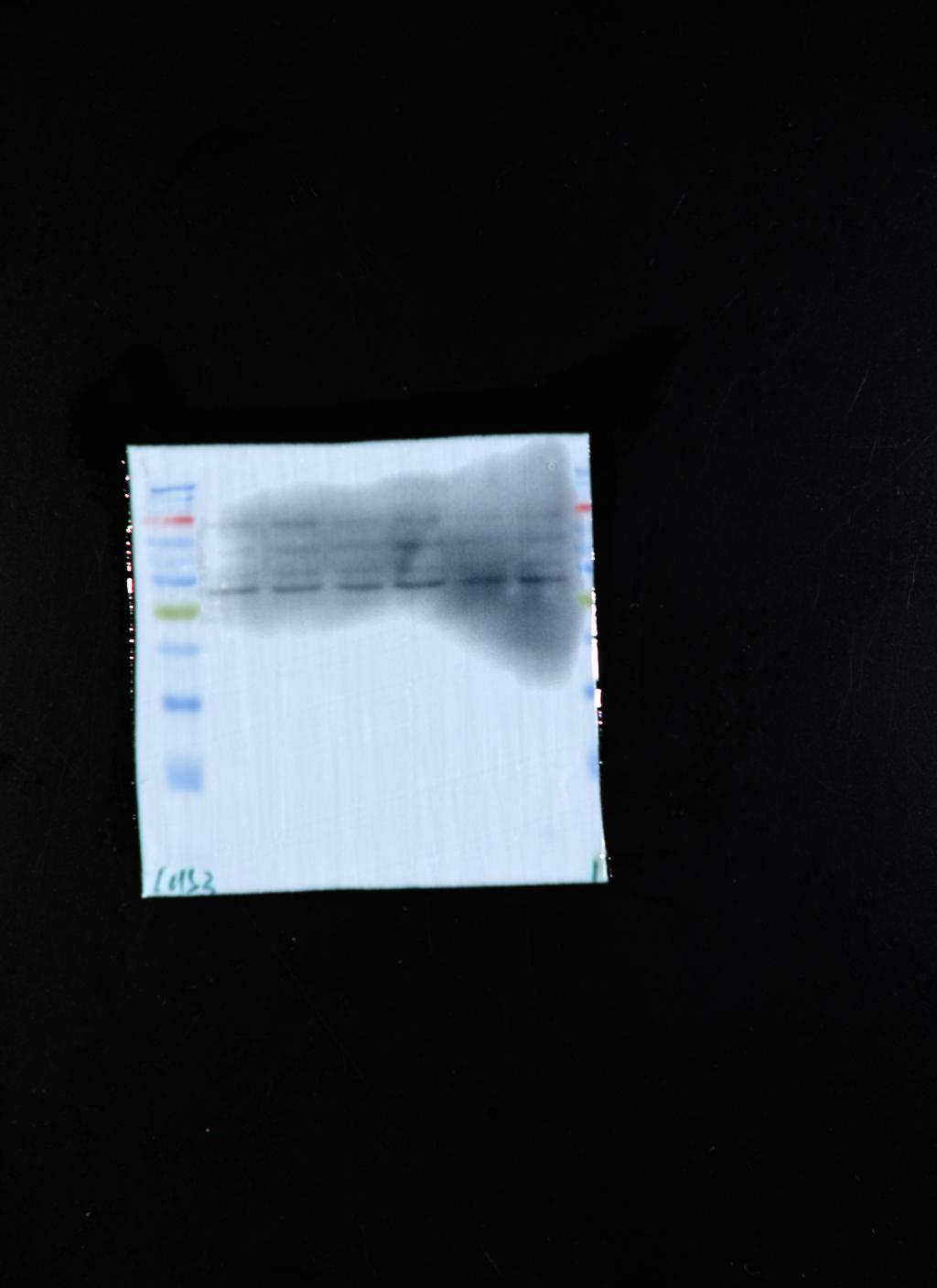

Supplement: Supplementary file 1 [file Data_Sheet_1.ZIP › Raw data/Figure 6/A. Cleaved Caspase3-a-Sham-CCI.jpg]

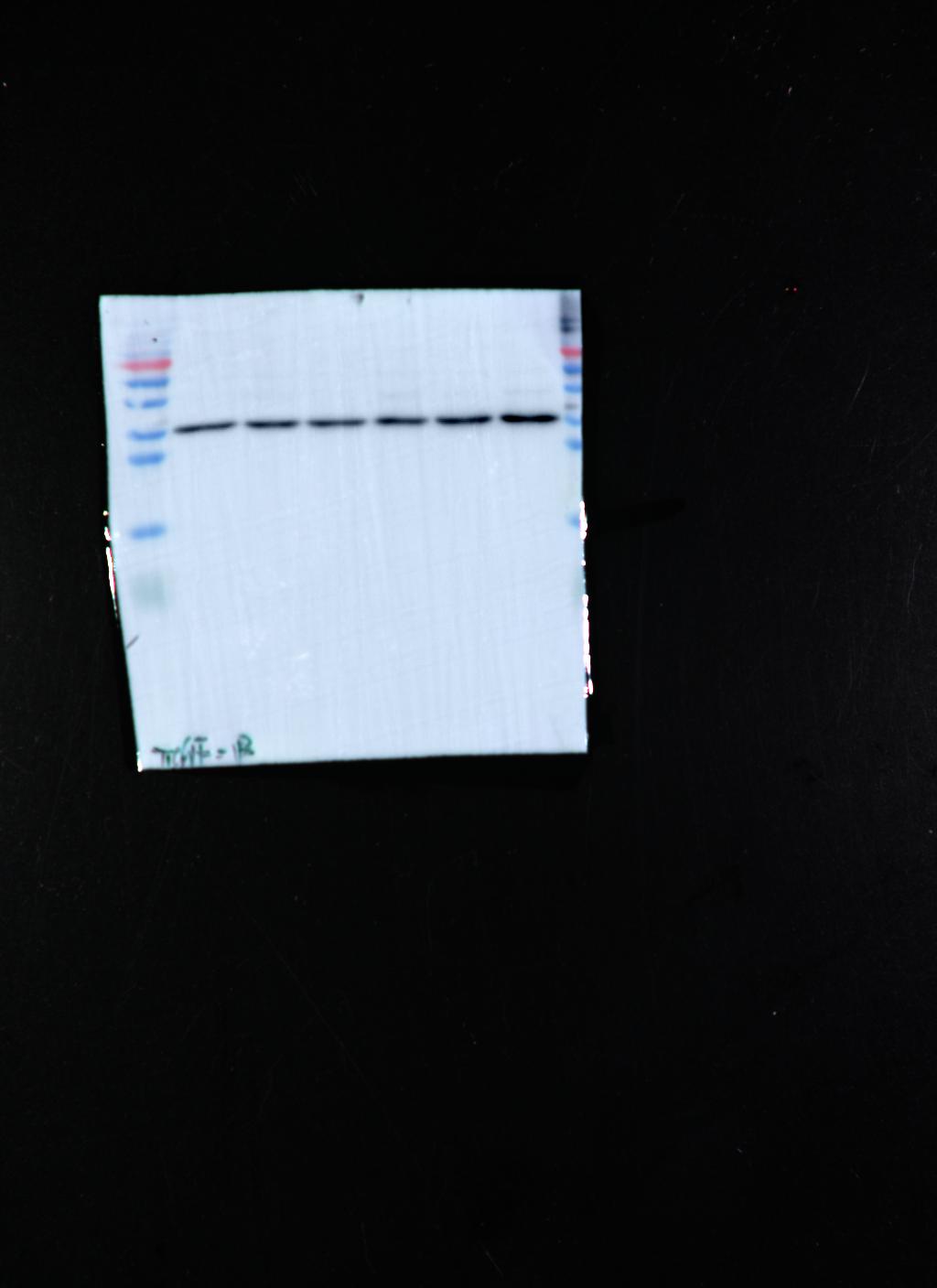

Supplement: Supplementary file 1 [file Data_Sheet_1.ZIP › Raw data/Figure 6/A. TGF-a┬ Sham-CCI.jpg]

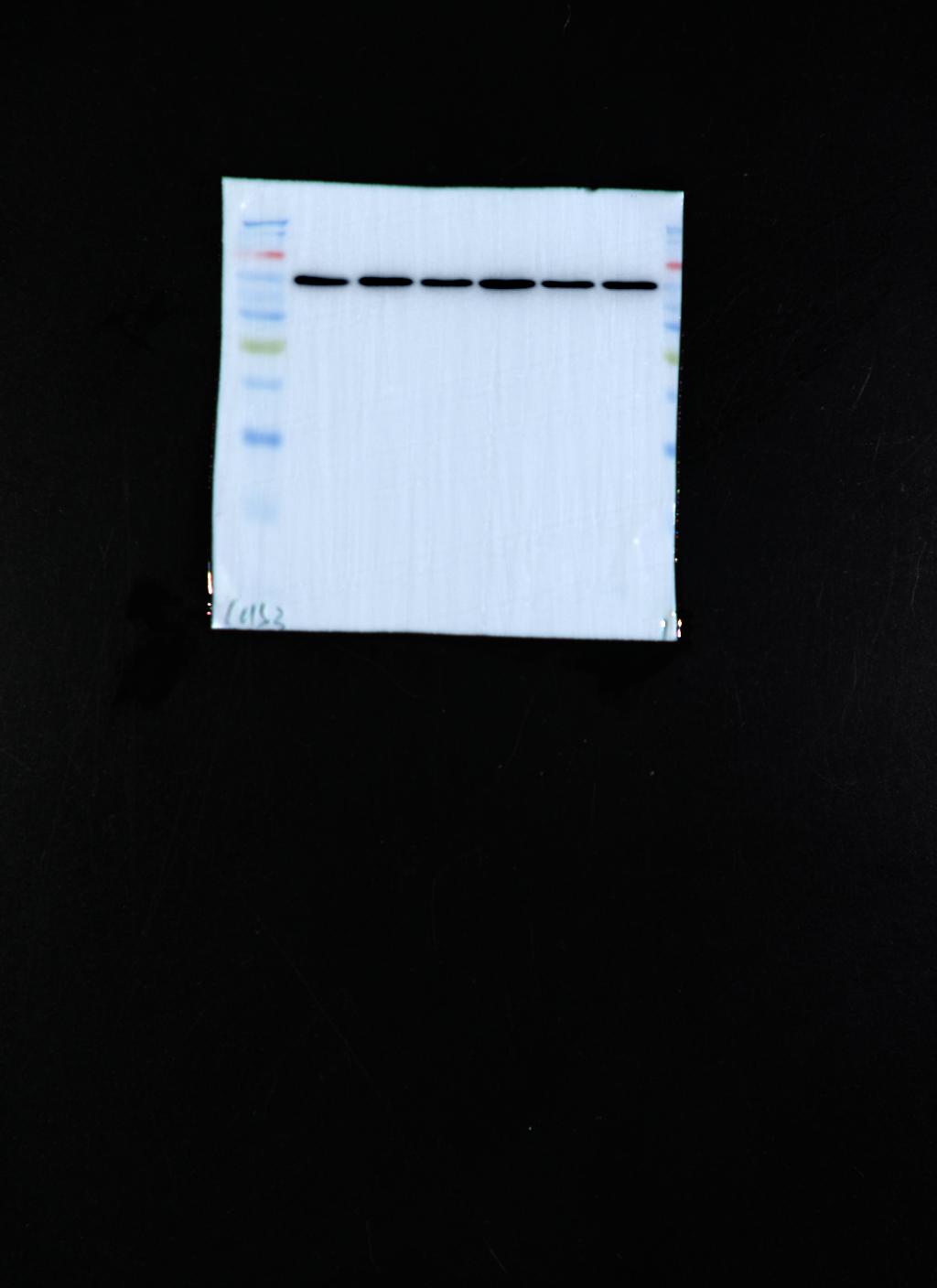

Supplement: Supplementary file 1 [file Data_Sheet_1.ZIP › Raw data/Figure 6/A. a-tubulin Sham-CCI.jpg]

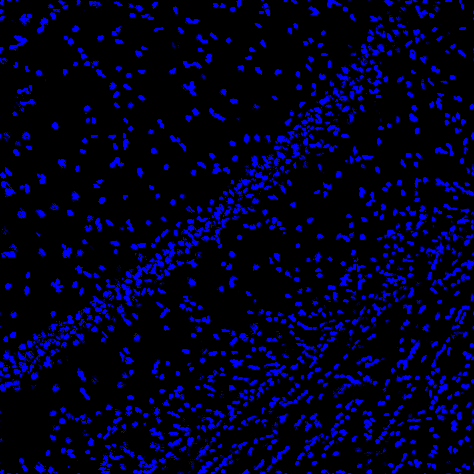

Supplement: Supplementary file 1 [file Data_Sheet_1.ZIP › Raw data/Figure 6/F. TUNEL staining/CCI-DAPI.tif]

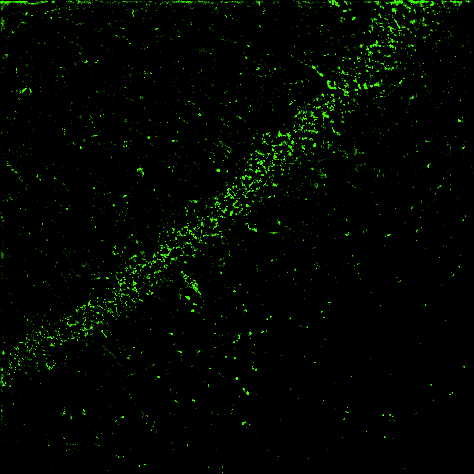

Supplement: Supplementary file 1 [file Data_Sheet_1.ZIP › Raw data/Figure 6/F. TUNEL staining/CCI-FITC.tif]

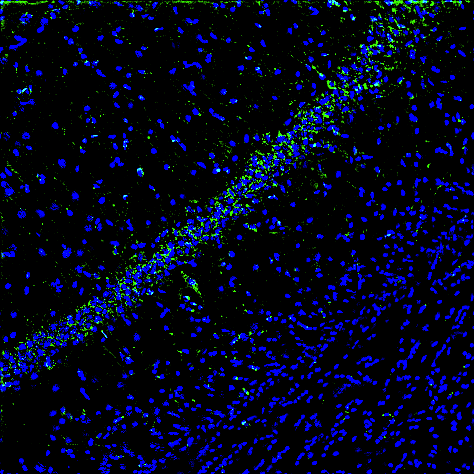

Supplement: Supplementary file 1 [file Data_Sheet_1.ZIP › Raw data/Figure 6/F. TUNEL staining/CCI-merge.tif]

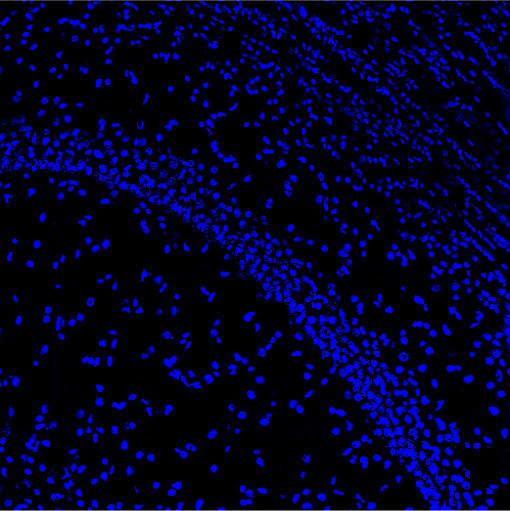

Supplement: Supplementary file 1 [file Data_Sheet_1.ZIP › Raw data/Figure 6/F. TUNEL staining/Sham-DAPI.tif]

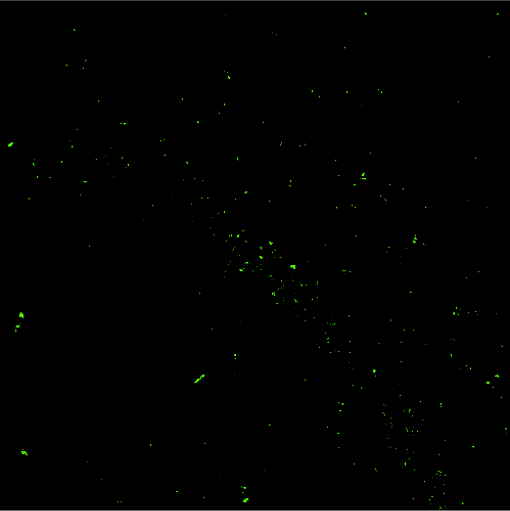

Supplement: Supplementary file 1 [file Data_Sheet_1.ZIP › Raw data/Figure 6/F. TUNEL staining/Sham-FITC.tif]

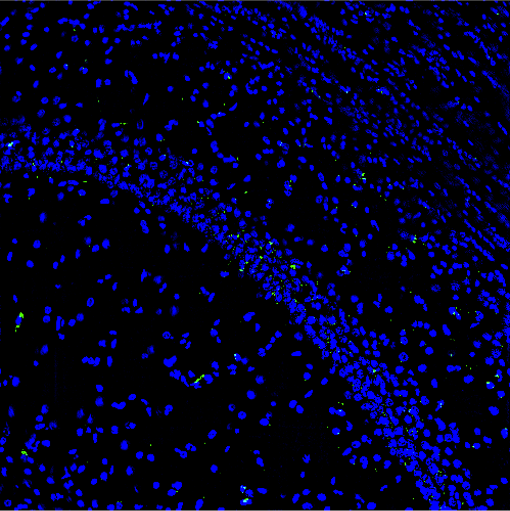

Supplement: Supplementary file 1 [file Data_Sheet_1.ZIP › Raw data/Figure 6/F. TUNEL staining/Sham-merge.tif]
