# Supplementary material for: Identification of potential key circular RNAs related to cognitive impairment after chronic constriction injury of the sciatic nerve
Source: Front Neurosci. 2022 Aug 18;16:925300. doi: 10.3389/fnins.2022.925300 (PMC9433970; doi:10.3389/fnins.2022.925300)
Supplement: Supplementary file 2 [file Data_Sheet_2.docx]

**Supplementary Materials for**

**Identification of potential key circular RNAs related to cognitive impairment after chronic constriction injury of the sciatic nerve**

**
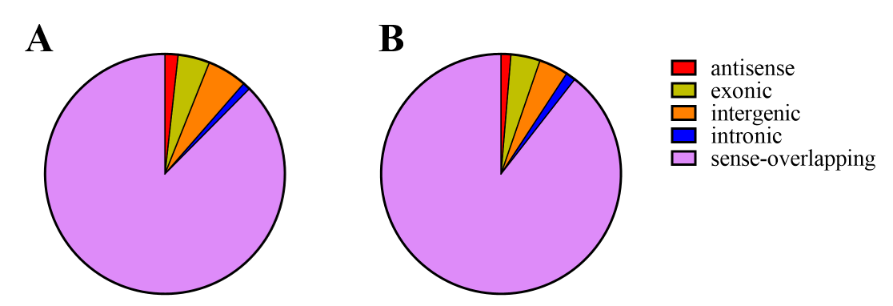
**

**Figure S1**. CircRNA category distribution of (A) the total circRNAs and (B) differentially expressed circRNAs.

**
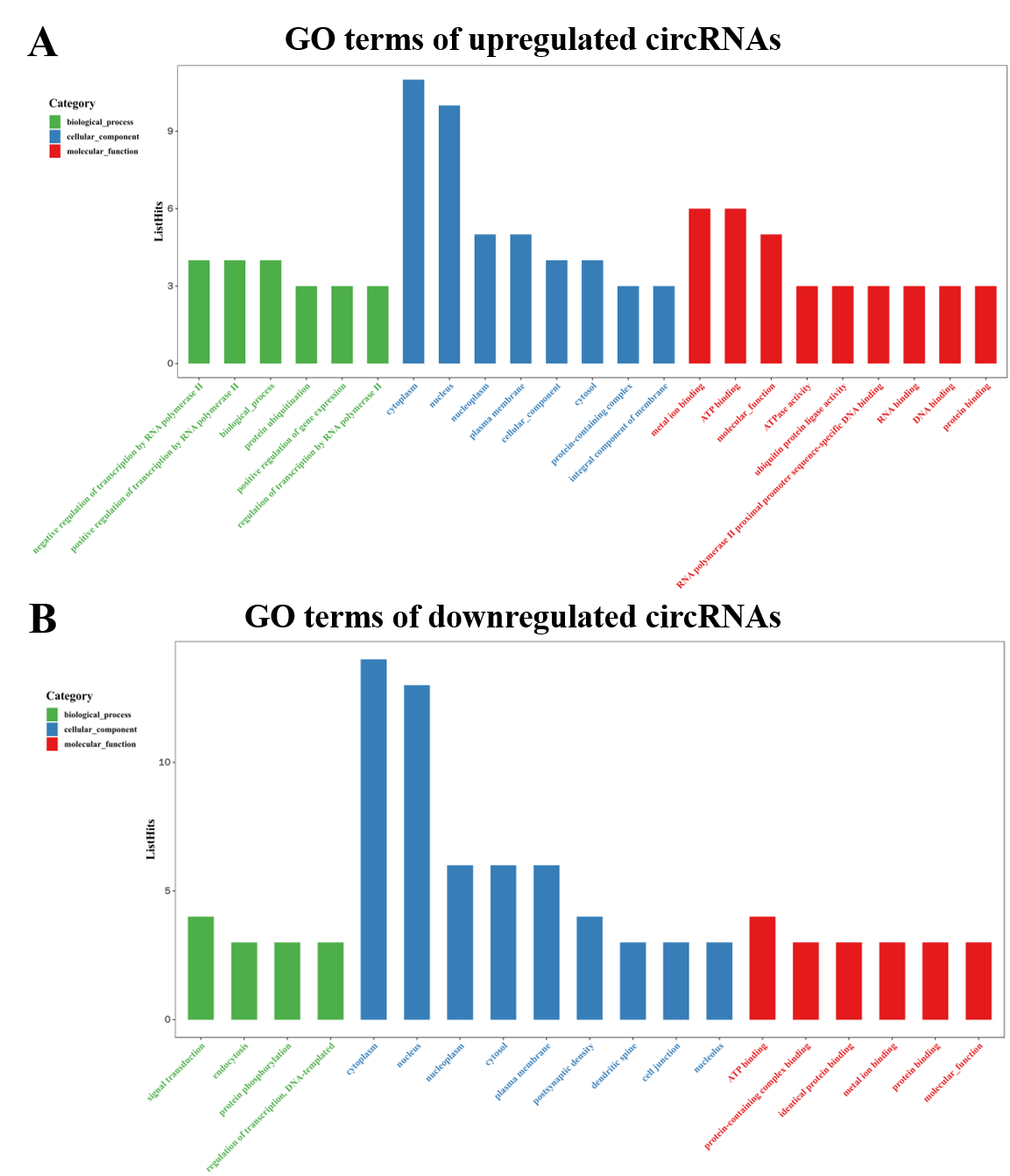
**

**Figure S2.** GO enrichment analysis showing the biological process (green), cellular component (blue), and molecular function (red) enriched by the target genes of the (A) upregulated and (B) downregulated differentially expressed circRNAs.
